# Integrative Mendelian Randomization and Pathomics Analysis Using Expression Quantitative Trait Loci and Genome‐Wide Association Study Data Identifies Mismatch Repair Genes as Prognostic Biomarkers in Gastric Adenocarcinoma

**DOI:** 10.1155/ijog/2686529

**Published:** 2026-03-09

**Authors:** Yingqiao Zhang, Dan Li, Yuyao Jin, Wenjuan Zhao, Pengyu Guo, Ziqi Wang, Xinyu Zhu, Zhenqi Ma, Lin Sui, Yanmeng Liang, Yang Liu, Xiushi Zhang

**Affiliations:** ^1^ Department of Radiology, Harbin Medical University Cancer Hospital, No. 150 Haping Road, Harbin, 150010, Heilongjiang, China, hrbmu.edu.cn

**Keywords:** gastric adenocarcinoma, histopathological image analysis, Mendelian randomization, mismatch repair genes, MutS homolog 2 (*MSH2*), tumor mutational burden

## Abstract

**Background:**

Mismatch repair (MMR) genes are implicated in stomach adenocarcinoma (STAD). This study assessed their causal role in STAD, prognostic value, and developed a histopathology‐based model to predict MutS homolog 2 (MSH2) expression.

**Methods:**

Using data from the IEU OpenGWAS database, five Mendelian randomization (MR) models evaluated causal links between MMR genes and gastric cancer (GC). Prognostic relevance was assessed via survival analysis. A random forest model using TCGA hematoxylin and eosin–stained images was trained to predict MSH2 expression. Biological insights were explored via pathomics score, gene set enrichment analysis (GSEA), immune infiltration, and tumor mutational burden (TMB).

**Results:**

MR analysis identified MLH1 and PMS2 as risk genes, while MSH2 had a protective effect. Cox regression confirmed MSH2 as an independent protective factor (HR = 0.690, 95% CI: 0.487–0.977, *p* < 0.05). The pathomics model predicted MSH2 expression with an AUC of 0.811. A comparison of high‐ and low‐survival‐probability (SP) groups showed differentially expressed genes, including SFRP4. The high‐SP group had elevated TMB and TP53 mutation frequency.

**Conclusion:**

MMR genes, especially MSH2, are critical in STAD development and prognosis. The image‐based model effectively predicts MSH2 expression, supporting the integration of genomic and histopathologic data for personalized GC care.

## 1. Introduction

Gastric cancer (GC) is a leading cause of cancer‐related mortality worldwide [1–4]. Its predominant histological subtype, gastric adenocarcinoma—also known as stomach adenocarcinoma (STAD)—constitutes more than 90% of GC cases. STAD is characterized by high biological heterogeneity, meaning that tumors can vary widely in their genetic, molecular, and cellular features. This diversity complicates the understanding of its pathogenesis and contributes to challenges in diagnosis and treatment [5]. Its aggressive nature, combined with a low early detection rate of approximately 20%, results in a poor 5‐year survival rate of less than 30% [6]. The International Agency for Research on Cancer reported more than 968,000 new GC cases and nearly 660,000 GC‐related deaths globally in 2022 [7]. Despite advances in therapeutic strategies, early diagnosis and accurate prognosis prediction for STAD remain major clinical challenges [8–11]. Therefore, there is an urgent need to explore and integrate current diagnostic and therapeutic modalities more effectively.

Precise assessment of the molecular features of tumors is crucial for overcoming these challenges. Advances in modern molecular imaging technologies have enabled the noninvasive, *in vivo* visualization of tumor‐specific targets. For instance, near‐infrared fluorescence/Cherenkov luminescence dual‐modal imaging can dynamically assess the expression levels of tumor programmed death‐ligand 1, providing a proof‐of‐concept for using imaging to infer the molecular biological status of tumors [12]. In clinical practice, image‐based evaluation has become a cornerstone of GC management. Specifically, the combination of enhanced computed tomography and serum tumor markers, such as alpha‐fetoprotein, has proven valuable in identifying specific subtypes like hepatoid adenocarcinoma of the stomach, highlighting the utility of integrating multimodal information to address specific clinical problems [13]. However, these methods primarily reflect the macroscopic phenotype or indirect indicators of tumors and still lack a direct, stable link to the core genomic functions that drive tumor development and progression.

Mismatch repair (MMR) genes play a crucial role in maintaining genomic stability by correcting DNA replication errors. Defects in these genes can lead to the accumulation of mutations and contribute to cancer development [14, 15]. Recent advances in molecular biology have elucidated the genetic background and molecular mechanisms underlying STAD, with MMR genes emerging as essential regulators in tumorigenesis [16–18]. MMR deficiency (MMRD), caused by MMR deletions, such as deletions in MutS homolog 1 (*MLH1*) and PMS1 homolog 2 (*PMS2*), is strongly associated with microsatellite instability (MSI), a condition that increases genomic instability and may drive the development and progression of GC [19]. Notably, MMRD has been identified as an independent and robust prognostic factor in advanced GC. However, there is limited efficacy of adjuvant 5‐fluorouracil chemotherapy for patients with MMRD, suggesting that MMR status influences chemotherapy response [20]. Furthermore, patients with MMR‐deficient gastroesophageal adenocarcinomas seem to benefit more from immunotherapy [21]. Despite these important findings, the precise roles of key MMR genes—including *MLH1*, MutS homolog 2 (*MSH2*), and *PMS2*—their causal relationships with STAD, and their impact on patient prognosis have yet to be fully understood [18, 22]. Current diagnostic methods for assessing MMR status, such as immunohistochemistry and polymerase chain reaction–based MSI testing, have limitations, underscoring the need for improved approaches.

Mendelian randomization (MR) is a powerful analytical method that uses genetic variants as instrumental variables (IVs) to minimize confounding and reverse causation biases common in observational studies [23–25]. This approach enhances the reliability of causal inference between target exposures and clinical outcomes [26]. Although contemporary genetic diagnostics often rely on liquid biopsy‐derived DNA analysis from peripheral blood and bodily fluids, these techniques are limited by invasive sampling procedures, high costs, long turnaround times, and insufficient ability to capture intratumoral heterogeneity [27]. Pathomics, which integrates machine learning with histopathological assessment, offers a novel solution by systematically quantifying tumor biological features and immune microenvironment alterations through computational analysis of cellular morphology and tissue architecture in histological specimens [28–30]. This approach has shown promising applicability in prognostic assessment of GC [31].

In this study, we aimed to elucidate the biological mechanisms of MMR in STAD and assess their clinical relevance. To achieve this, we integrated genetic causal inference with advanced computational pathology approaches, leveraging MR analysis to infer causal relationships with a pathomics‐based model to comprehensively investigate the biological mechanisms of MMR in STAD and quantitatively characterize tumor features. This combined strategy addresses current knowledge gaps and has the potential to improve early diagnosis, optimize treatment strategies, and enhance prognosis prediction, ultimately contributing to more precise and personalized management of gastric adenocarcinoma.

## 2. Materials and Methods

### 2.1. Association Between MMR‐Related Genes and GC

#### 2.1.1. Study Design and Data Sources

We conducted an MR study to investigate the causal relationship between MMR‐related genes and GC risk. Expression quantitative trait loci (eQTLs) for MMR genes served as exposures, single nucleotide polymorphisms (SNPs) significantly associated with these genes served as IVs, and genome‐wide association study (GWAS) summary statistics for GC were used as the outcome variable. Both datasets were derived from European ancestry populations. The overall design is illustrated in Figure S1, and detailed dataset characteristics are provided in Table S5 of the Supporting Information.

#### 2.1.2. Selection of IVs

IVs were selected based on their association with gene expression and independence from confounders. Specifically, IVs were required to satisfy three core assumptions of MR:

(1) Relevance: IVs were strongly correlated with the exposure, using a significance threshold of *p* *< *1 × 10^−5^. Although this threshold is less stringent than the conventional genome‐wide significance level (*p* < 5 × 10^−8^), it balances instrument strength and availability. (2) Independence: IVs were independent of confounders affecting both exposure and outcome. (3) Exclusion restriction: IVs influenced the outcome only through the exposure, with no horizontal pleiotropy.

SNPs were pruned for linkage disequilibrium (LD) and filtered to exclude variants associated with confounders or the outcome. Palindromic and outlier SNPs were removed to minimize bias. eQTL data for MMR‐related genes (*MSH2*, *MLH1*, mutL homolog 3 *[MLH3]*, MutS homolog 3 *[MSH3]*, MutS homolog 6 *[MSH6]*, PMS1 homolog 1 *[PMS1]*, PMS2) and GWAS summary statistics for GC (633 cases and 174,006 controls; dataset: finn‐b‐C3_STOMACH_EXALLC) were obtained from the Integrative Epidemiology Unit Open Genome‐Wide Association Studies (IEU OpenGWAS) project (https://gwas.mrcieu.ac.uk/), accessed on October 29, 2024. Both datasets are derived from populations with European ancestry, which may limit the generalizability of findings to other ethnic groups. Detailed characteristics of the datasets are summarized in Table S5 of the Supporting Information. Ethical approval was not required for this secondary analysis, as all original studies obtained informed consent from the participants. Detailed criteria for IV selection, including all specific thresholds and parameters, are described in the Supporting Information S2.1.2.

#### 2.1.3. MR Analysis

MR analysis was conducted using five regression methods, with inverse‐variance weighted (IVW) as the primary approach. Tests for heterogeneity and horizontal pleiotropy were conducted to validate IV assumptions. Sensitivity analyses, including leave‐one‐out tests, assessed robustness. Software tools and detailed statistical thresholds are provided in Supporting Information S2.1.3.

The five regression methods used were MR‐Egger regression, IVW, weighted median, weighted mode, and simple mode. Because of its efficiency under valid IV assumptions, IVW was the primary method, while the others provided complementary analyses. Cochran’s Q‐test assessed heterogeneity among SNPs, with *p* < 0.05 indicating significant heterogeneity. Horizontal pleiotropy was evaluated using the MR‐Egger intercept test and the MR‐PRESSO global test; nonsignificant differences between the intercept term of the MR‐Egger regression and 0 (*p* > 0.05) and *p* > 0.05 in MR‐PRESSO indicate an absence of horizontal pleiotropy.

Leave‐one‐out sensitivity analysis was performed by sequentially removing each SNP to assess its influence on the overall estimate. All analyses were implemented using the TwoSample MR package in R Version 4.1.0, with a significance threshold of *α* = 0.05.

### 2.2. Survival and Prognostic Analysis of MMR‐Related Genes

Survival analyses focused on genes with significant causal effects on GC risk that were identified through MR (“positive genes”). Kaplan–Meier (KM) curves, log‐rank tests, and Cox proportional hazards regression models (univariate and multivariate) were used to evaluate overall survival (OS) and prognostic significance. Subgroup and interaction analyses were performed to explore clinical heterogeneity. Detailed statistical methods and software implementations are available in Supporting Information S2.2.

### 2.3. Establishment of the Pathomics Model

#### 2.3.1. Image Acquisition, Segmentation, and Feature Extraction

Histopathological images were processed to extract quantitative features for modeling gene expression. Two experienced pathologists, blinded to all molecular subtypes and patient outcomes, independently reviewed the whole‐slide images. They selected representative, high‐quality tumor regions based on predefined criteria (e.g., tissue integrity, absence of artifacts, and sufficient tumor cellularity). A fixed number of subimages were then extracted from these consensus regions for subsequent feature extraction. Detailed protocols for image acquisition, segmentation, and feature extraction are described in Supporting Information S2.3.1.

#### 2.3.2. Establishment and Evaluation of the Random Forest Model

A random forest classifier was developed to predict gene expression levels using selected histopathological features. The dataset was split into training and validation sets, and feature selection was performed in two stages. Model performance was evaluated using accuracy, sensitivity, specificity, AUC, calibration, and decision curve analysis (DCA). Detailed protocols for the establishment and evaluation of the random forest model are described in Supporting Information S2.3.2.

#### 2.3.3. Pathomics Score (PS) for Survival and Prognosis Analysis

The PS is defined as a continuous, quantitative output from the trained pathomics model. For each patient’s H&E image, the PS represents the model’s computed probability or likelihood score for the sample belonging to the “high‐risk” molecular phenotype (e.g., associated with low MMR gene expression). A higher PS indicates a greater predicted probability of this phenotype. To assess the clinical relevance of the PS, patients were stratified into high‐ and low‐PS groups using an optimal cutoff value determined by maximally selected rank statistics (survminer R package). Baseline clinical characteristics were summarized and compared between the two groups using appropriate statistical tests (chi‐square test for categorical variables; Student’s *t*‐test or Wilcoxon rank‐sum test for continuous variables). The prognostic value of the PS groups was evaluated through KM survival analysis with log‐rank tests, univariate and multivariate Cox proportional hazards regression, as well as subgroup analyses and interaction tests. In the Cox models, continuous variables (e.g., age) were treated as continuous predictors, while categorical variables (e.g., tumor stage, gender) were included as factors. The proportional hazards assumption was verified using Schoenfeld residual plots, and no significant violations were observed for the primary variables. Detailed descriptions of the statistical methods and subgroup analyses are provided in Supporting Information S2.3.3.I.

#### 2.3.4. Establishment and Evaluation of the Nomogram Model

A Cox regression nomogram incorporating the PS and clinical variables was constructed to predict survival probabilities (SPs) at multiple time points. Model discrimination, calibration, and clinical utility were assessed.

The nomogram was developed to predict 36‐, 48‐, and 60‐month SPs, incorporating the PS and six clinically relevant variables selected based on univariate analysis and clinical judgment. Time‐dependent ROC curves were generated using the R package timeROC to evaluate model discrimination at each time node (36, 48, and 60 months).

Calibration of the nomogram prediction model was assessed with plots of the calibration curves. The clinical net benefit was evaluated by DCA. The predictive performance of the timeAUC of the PS‐clinical nomogram was compared to models constructed from individual clinical variables using statistical tests to evaluate differences in AUC. A detailed description of the nomogram design and validation methods is provided in Supporting Information S2.3.4.

### 2.4. Exploration of the Biological Relevance of the Nomogram Model

#### 2.4.1. Differential Gene Expression and Enrichment Analysis Between SP Groups

Differential expression analysis was performed between high‐ and low‐SP groups predicted by the nomogram. Gene set enrichment analyses identified biological pathways associated with survival differences. Based on the timeAUC value predicted by the PS‐clinical nomogram prediction model, the SP at the time point with the highest timeAUC was divided into high and low groups using the median value of the binary variable of SP, as determined using the median method. RNAseq data (HTSeq–PKM) from the STAD cohort within The Cancer Genome Atlas (TCGA–STAD) project were log2 transformed. Differentially expressed genes (DEGs) between SP groups were identified using thresholds of fold change > 1.2 and *p* < 0.05, without applying multiple testing correction, to avoid potentially masking biologically relevant signals [32]. The differential expression analysis was performed as an exploratory, hypothesis‐generating step to inform subsequent gene set enrichment analysis (GSEA). Therefore, a nominal *p* value threshold (*p* < 0.05) was used without multiple testing correction, with the understanding that individual gene‐level results should be interpreted cautiously. The primary mechanistic insights were derived from the more robust pathway‐level GSEA results.

The top 20 most significantly downregulated genes were visualized using heatmaps generated with the R package ggplot2 (v3.5.2) [33]. GSEA was performed using the R package clusterProfiler (v4.6.2) with Kyoto Encyclopedia of Genes and Genomes (KEGG; c2.cp.kegg.v7.5.1.symbols.gmt) and Hallmark (h.all.v7.5.1.symbols.gmt) gene sets from the Molecular Signatures Database (MSigDB) to explore biological pathways associated with differences in SP expression between high and low groups.

#### 2.4.2. Correlation Between SP and Immune Cell Abundance

Immune cell infiltration levels were estimated from gene expression data, and correlations with SP were assessed. Gene expression data from GC samples were uploaded to the ImmuCellAI database (https://guolab.wchscu.cn/ImmuCellAI/#!/) to estimate immune cell infiltration levels. Spearman’s rank correlation was used to assess the correlations between SP and immune cell abundance. Detailed methods for immune cell estimation and statistical analyses are provided in Supporting Information S2.4.2.

#### 2.4.3. Tumor Mutational Burden (TMB) and Somatic Mutation Analysis

The TMB and somatic mutation profiles were analyzed to explore genomic differences between survival groups. Mutation annotation format (.MAF) files from the TCGA–STAD cohort were downloaded. The TMB was calculated as the number of somatic mutations per megabase using established pipelines and the R package maftools. Differences in TMB between high‐ and low‐SP groups were assessed using the Wilcoxon rank‐sum test. Somatic variants were stored in the files with the MAF extension, and somatic mutation profiles were analyzed and visualized using the R package maftools, highlighting the top 15 most frequently mutated genes in the STAD cohort. Detailed methodologies for mutation data processing and statistical comparisons are provided in Supporting Information S2.4.3.

## 3. Results

### 3.1. Causal Association Between MMR‐Related Genes and Stomach Cancer

**FIGURE 1:**
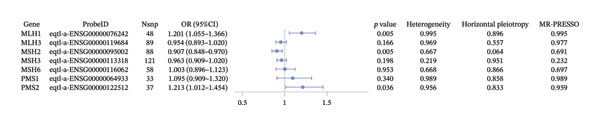
Forest plot of the causal association of MMR‐associated genes with gastric cancer.

Using eQTL data of protein‐coding MMR‐related genes (*MSH2*, *MLH1*, *MLH3*, *MSH3*, *MSH6*, *PMS1*, and *PMS2*), 474 SNPs associated with GC were screened as IVs (Table S1 in the Supporting Information). MR analysis was performed on these SNPs to explore the causal association between MMR‐related genes and GC risk (Figure 1).

IVW analysis revealed that three MMR‐related genes (*MLH1*, *MSH2*, and *PMS2*) had significant causal associations with GC. *MLH1* (odds ratio [OR] = 1.201, 95% confidence interval [CI]: 1.055–1.366, *p* = 0.005) and *PMS2* (OR = 1.213, 95% CI: 1.012–1.454, *p* = 0.036) were associated with increased GC risk, whereas *MSH2* (OR = 0.907, 95% CI: 0.848–0.970, *p* = 0.005) was associated with decreased GC risk.

Heterogeneity tests indicated no significant heterogeneity among the SNPs of the three MMR‐related genes (*I* ^2^ < 50%, Cochran’s *Q* test: *p* > 0.05). MR‐Egger intercept tests showed no significant difference between the intercept term and 0 (*p* > 0.05), and MR‐PRESSO global tests showed no evidence of horizontal pleiotropy (*p* > 0.05) (Table S2 in the Supporting Information). Funnel plots were evenly distributed and symmetrical, and the leave‐one‐out analysis did not identify any influential SNPs that could have significantly affected the MR results (Figure S2 in the Supporting Information), supporting the robustness of the MR findings.

### 3.2. Prognostic Significance of MLH1, MSH2, and PMS2

Expression cutoff values for *MLH1* (2.045), *MSH2* (1.744), and *PMS2* (1.702) were determined using the R package survminer. Patients were stratified into high‐ and low‐expression groups. Median OS times for high‐ versus low‐expression groups of *MLH1*, *MSH2*, and *PMS2* were 26.0 versus 56.2, 56.2 versus 26.7, and 34.8 versus 29.0 months, respectively.

Log‐rank tests showed significant OS differences for *MLH1* and *MSH2* high‐ and low‐expression groups (*p* < 0.05), which were consistent with the direction of MR. No significant difference in OS was observed for high‐ and low‐expression *PMS2* groups (*p* = 0.165). These results suggest that high *MLH1* expression is associated with poorer OS, whereas high *MSH2* expression correlates with improved OS (Figures 2(a), 2(b), 2(c)).

FIGURE 2(a–c) Kaplan–Meier (KM) overall survival (OS) curves for *MLH1*, *MSH2*, and *PMS2*. (d) Subgroup analysis of *MSH2*.

**Figure:**
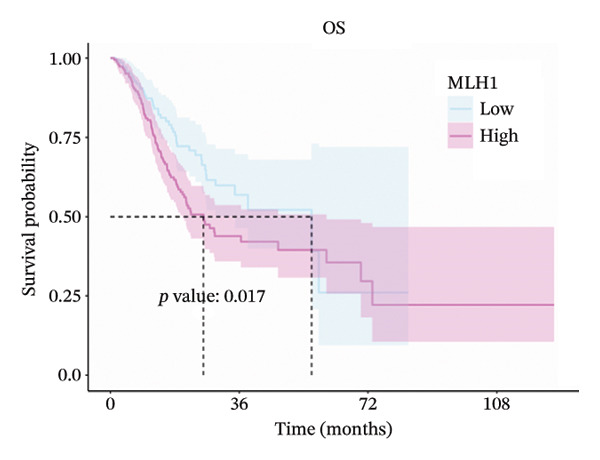
(a)

**Figure:**
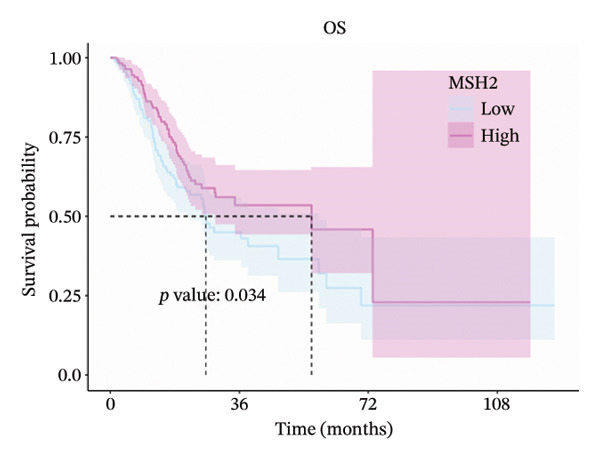
(b)

**Figure:**
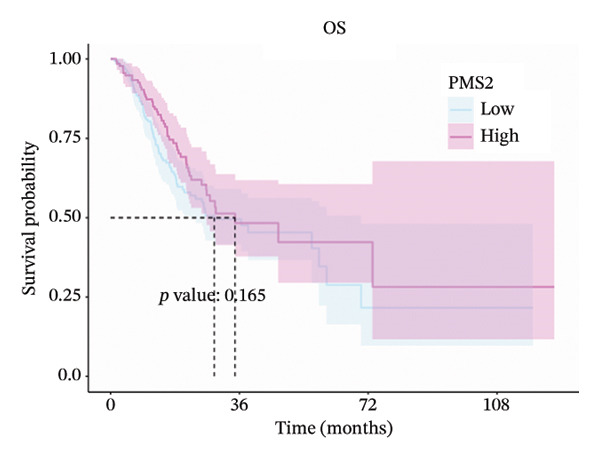
(c)

**Figure:**
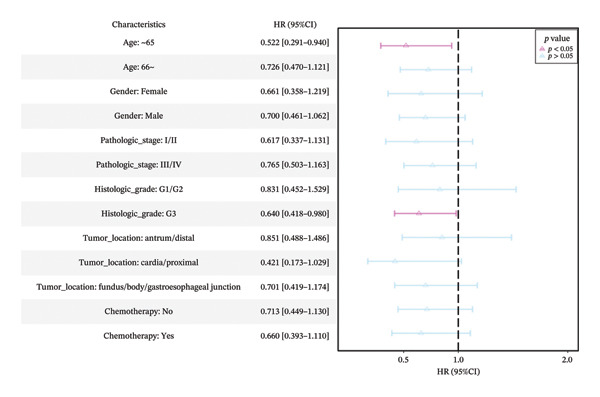
(d)

Univariate Cox regression identified *MSH2* (HR = 0.691, 95% CI: 0.490–0.975, *p* < 0.05) and *PMS2* (HR = 0.780, 95% CI: 0.549–1.108, *p* > 0.05) as protective factors for OS, whereas high expression of *MLH1* was associated with increased risk (HR = 1.553, 95% CI: 1.079–2.233, *p* < 0.05). The directions of the HRs for *MSH2* and *MLH1* were consistent with their respective KM survival curves. Although the KM curve suggested a protective effect for *PMS2*, it was opposite to the direction observed in the MR analysis.

Notably, *MLH1* is widely recognized as a tumor suppressor gene within the MMR family, serving as a protective factor for many cancers, with numerous studies demonstrating that tumor development is often associated with the loss or decreased expression of MMR genes [34–36]. Multivariate Cox regression analysis adjustment revealed that high expression of *MSH2* is an independent protective factor for OS (HR = 0.690, 95% CI: 0.487–0.977, *p* < 0.05). Based on these findings, the *MSH2* gene was selected as the representative biomarker of the MMR gene family for further analyses in STAD.

Subgroup analyses showed that increased *MSH2* expression was a significant protective factor for OS in the patients aged ≤ 65 years (HR = 0.522, 95% CI: 0.291–0.940, *p* < 0.05) and those with histologic grade G3 tumors (HR = 0.640, 95% CI: 0.418–0.980, *p* < 0.05). Although not statistically significant, elevated *MSH2* expression showed a protective trend in patients aged > 65 years (HR = 0.726, 95% CI: 0.470–1.121, *p* > 0.05) and in histologic grades G1/G2 (HR = 0.831, 95% CI: 0.452–1.529, *p* > 0.05) (Figure 2(d)). Interaction tests yielded *p* values of 0.455 and 0.526 for age and histologic grade subgroups, respectively (Table S3 in the Supporting Information), indicating no significant interaction between *MSH2* and different age subgroups and different histologic grade subgroups. In other words, the protective effect of *MSH2* on OS was similar across these subgroups. Similar trends were observed for other clinical covariates, though without statistical significance. Baseline characteristics of the 328 GC patients included in the survival analysis from the TCGA database are summarized in Table S7 of the Supporting Information.

### 3.3. Construction and Evaluation of the MSH2 Pathomics Model

Our previous results identified *MSH2* expression levels as an independent prognostic factor in GC. Therefore, we constructed a pathomics model to predict *MSH2* expression levels in GC patients based on hematoxylin and eosin (H&E)–stained histopathological images.

The TCGA dataset was randomly divided into a training set (*n* = 204) and a validation set (*n* = 87). Baseline characteristics between these sets and the validation set showed no significant differences (*p* > 0.05), confirming comparability between the two groups. Using mRMR combined with recursive feature elimination (RFE) and the RF algorithm, mRMR_RFE screening was performed. The nine optimal image features were identified and used to calculate the corresponding PS (Figures 3(a), 3(b)).

FIGURE 3(a–b) The nine selected features and their relative importance in the random forest algorithm used to predict *MSH2* expression levels based on histopathology image features. (c–d) Area under the receiver operating characteristic curve (AUC) for the model’s prediction of *MSH2* expression in the training and validation sets. (e–f) Calibration curves comparing predicted probabilities with the true value of the model for MSH2 expression status in the training and validation sets. (g–h) Decision curve analysis (DCA) curves of the model in the training and validation sets. (i–j) Wilcoxon test to compare whether pathomics scores are different between high and low gene groups. ns, *p* ≥ 0.05; ^∗^ *p* < 0.05; ^∗∗^ *p* < 0.01; ^∗∗∗^ *p* < 0.001.

**Figure:**
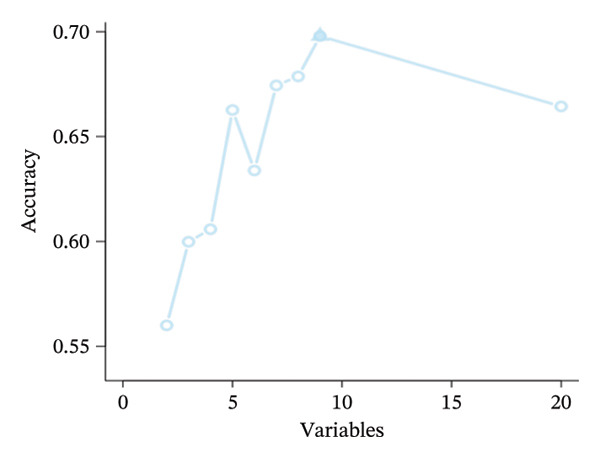
(a)

**Figure:**
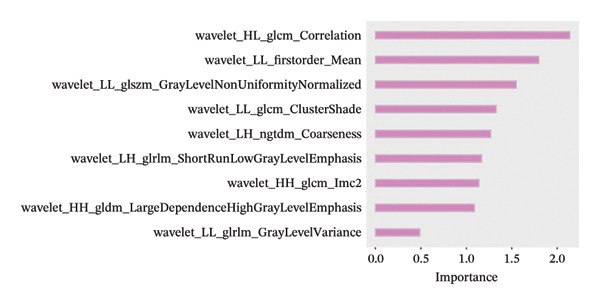
(b)

**Figure:**
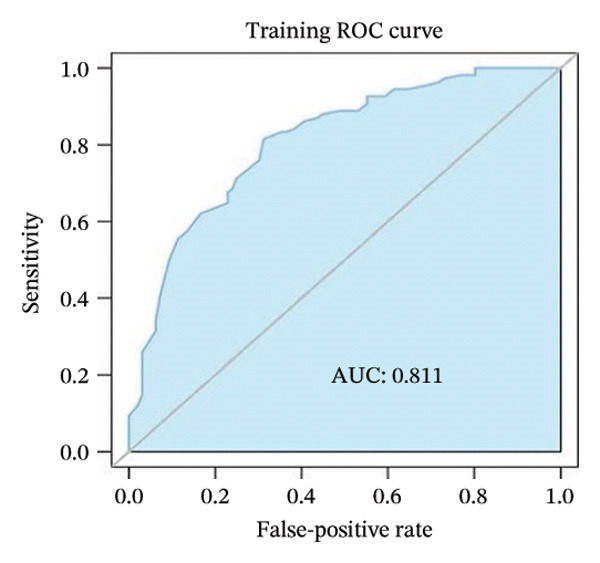
(c)

**Figure:**
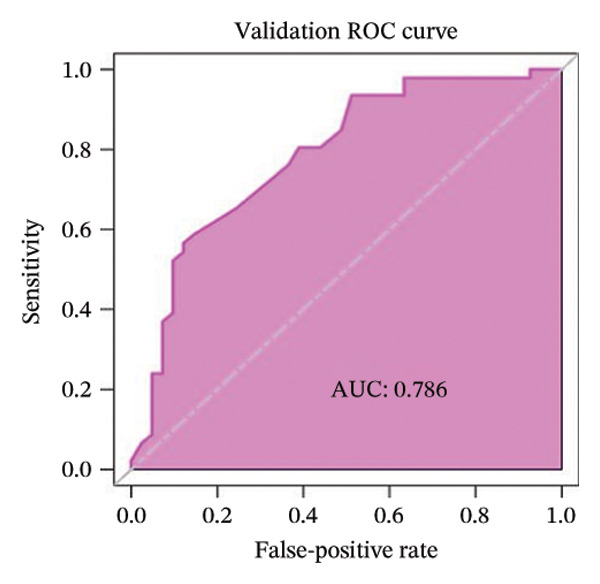
(d)

**Figure:**
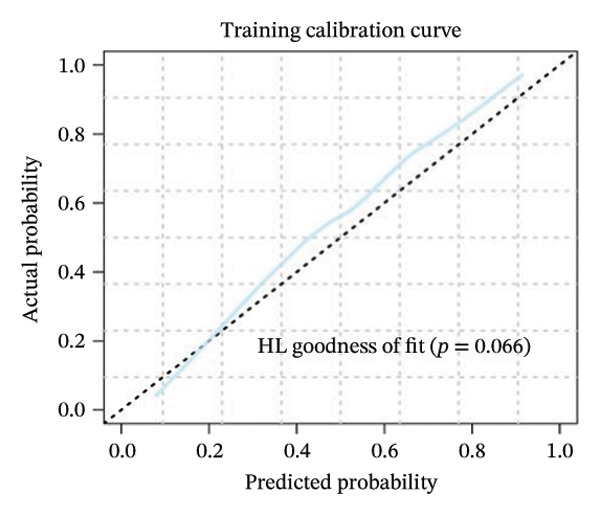
(e)

**Figure:**
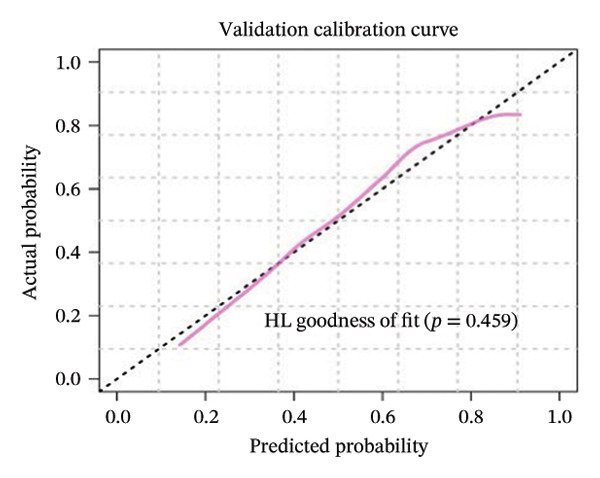
(f)

**Figure:**
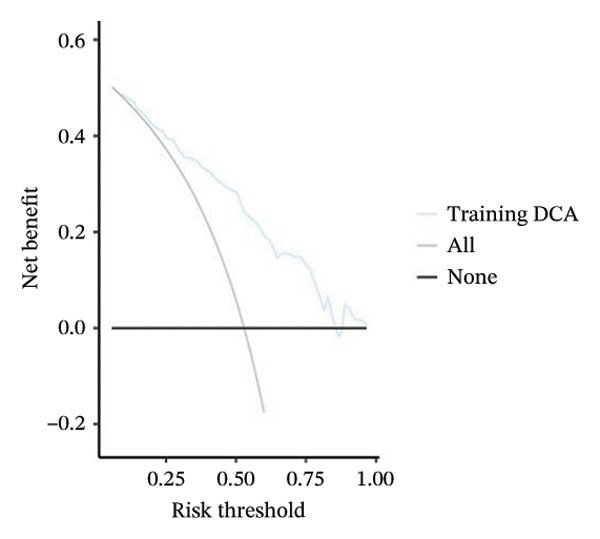
(g)

**Figure:**
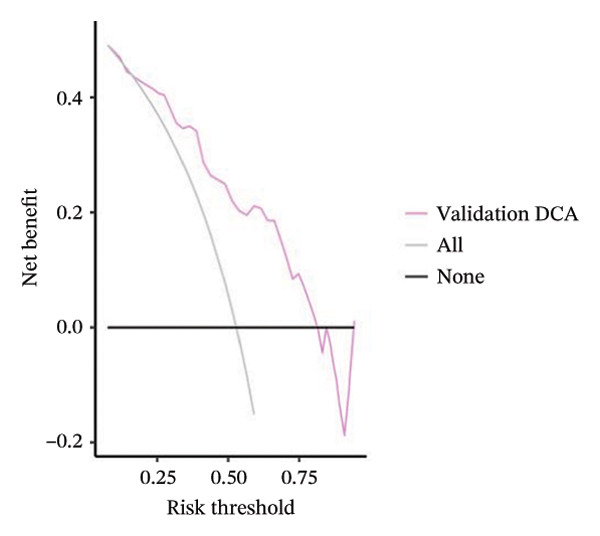
(h)

**Figure:**
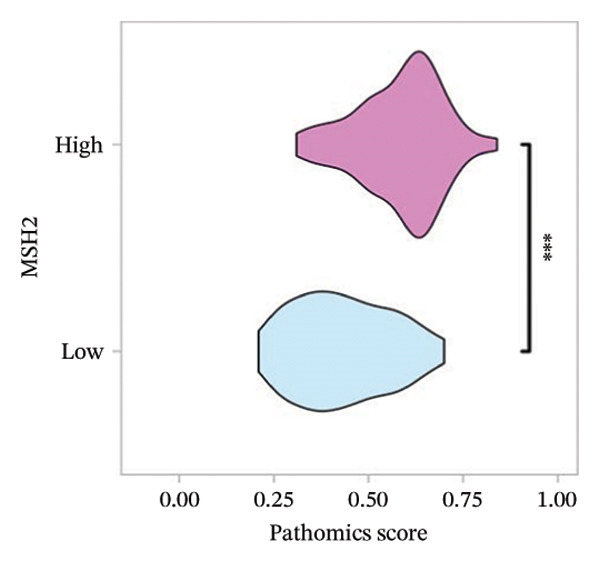
(i)

**Figure:**
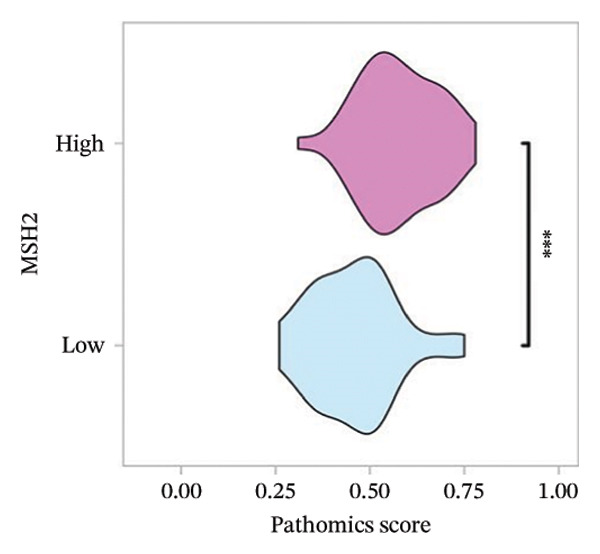
(j)

Model performance was robust, with an AUC of 0.811 in the training set and 0.786 in the validation set. Calibration curves and the Hosmer–Lemeshow goodness‐of‐fit test indicated the pathological omics prediction model achieved excellent agreement between the predicted probability of elevated gene expression and the true value (*p* > 0.05). DCA demonstrated the model’s significant clinical utility (Figures 3(c), 3(d), 3(e), 3(f), 3(g), 3(h)).

Key metrics for the training set included a threshold of 0.495, accuracy of 0.755, sensitivity of 0.815, specificity of 0.688, and Brier score of 0.194. The validation set showed accuracy of 0.713, sensitivity of 0.565, specificity of 0.878, and Brier score of 0.205 (Table S8 in the Supporting Information). The model demonstrates good specificity and reasonable overall accuracy, collectively indicating the model’s potential to achieve good clinical predictive performance.

Additionally, the Wilcoxon rank‐sum tests confirmed that the PS was significantly high in the high *MSH2* expression group compared to the low *MSH2* group (*p* < 0.05), demonstrating good discrimination ability of the pathomics model (Figures 3(i), 3(j)).

### 3.4. Prognostic Value of PS

Using a cutoff value of 0.540, patients were stratified into the high‐PS group (*n* = 126) and low expression group (*n* = 165) (Table S9 in the Supporting Information). Except for histologic type (*p* = 0.002), no significant differences were observed in the distribution of age and other clinical factors between the high‐ and low‐PS groups (*p* > 0.05). Median survival time was significantly longer in the high‐PS group compared to the low‐PS group (56.2 vs. 26.067 months). High‐PS expression was significantly associated with improved OS (*p* = 0.02) and serves as a protective factor for OS in both univariate and multivariate Cox analyses (HR = 0.644, 95% CI: 0.442–0.937, *p* = 0.021) and (HR = 0.671, 95% CI: 0.455–0.991, *p* = 0.045) (Figures 4(a), 4(b)).

FIGURE 4Cox univariate (a), multivariate analysis (b) of the pathomics score (PS) and subgroup analysis of PS (c).

**Figure:**
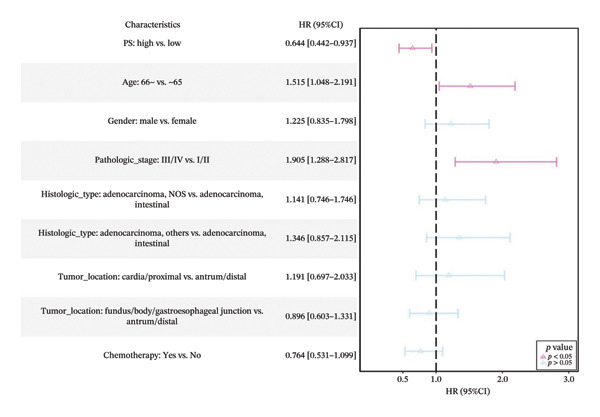
(a)

**Figure:**
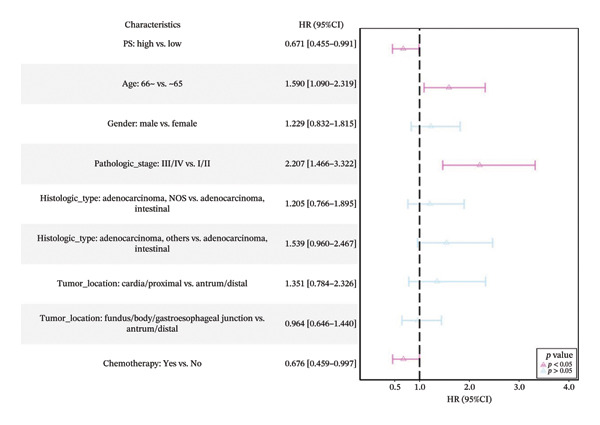
(b)

**Figure:**
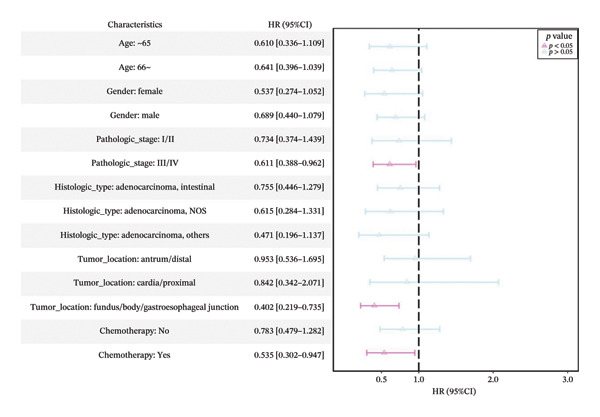
(c)

Subgroup analyses showed that elevated PS was a protective factor for OS in patients aged ≤ 65 years (HR = 0.610, 95% CI: 0.336–1.109, *p* = 0.11) and > 65 years (HR = 0.641, 95% CI: 0.396–1.039, *p* = 0.071), with no significant interaction between PS and different age subgroups (*p* = 0.85). Similar protective trends were observed across other covariates, with consistent effects across subgroups (Figure 4(c); Table S4 in the Supporting Information). These results support PS, reflecting *MSH2* expression, as an independent prognostic marker in GC.

### 3.5. Model Establishment of the Nomogram

A nomogram integrating PS and six clinical variables was constructed based on the Cox regression model (Figure 5(a)). Time‐dependent ROC analysis showed AUCs of 0.684, 0.736, and 0.719 for predicting 36‐, 48‐ and 60‐month OS, respectively. Calibration curves demonstrated good agreement between predicted and observed SPs, and DCA confirmed the model’s clinical usefulness (Figures 5(b), 5(c), 5(d)).

FIGURE 5(a) Nomograms of 36‐, 48‐, and 60‐month survival probabilities plotted by Cox regression for the pathomics score (PS), and six clinical variables. (b–d) Calibration curve and decision curve analysis (DCA) curves of the nomogram model.

**Figure:**
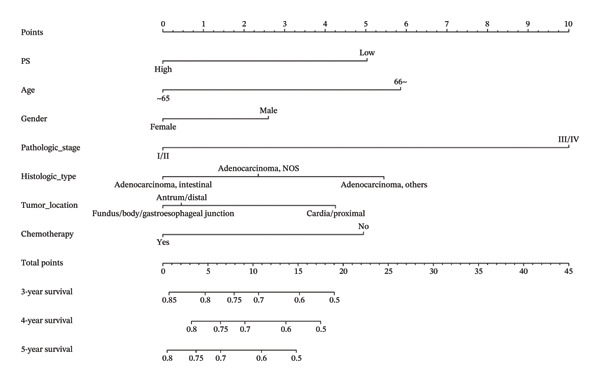
(a)

**Figure:**
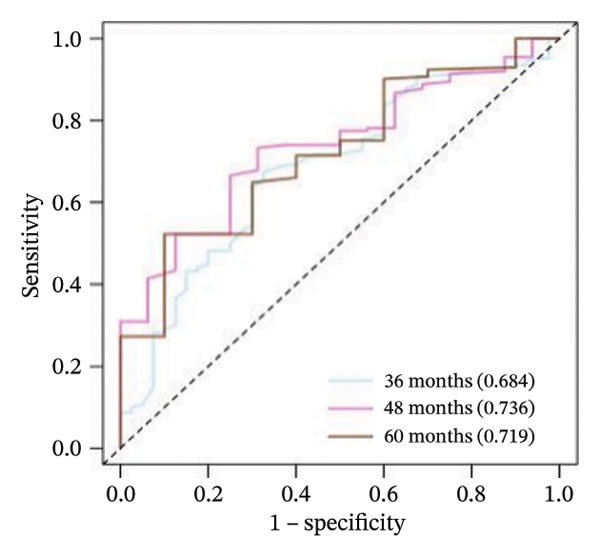
(b)

**Figure:**
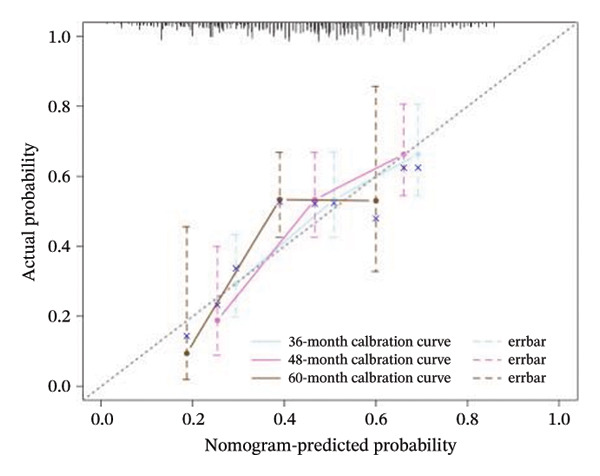
(c)

**Figure:**
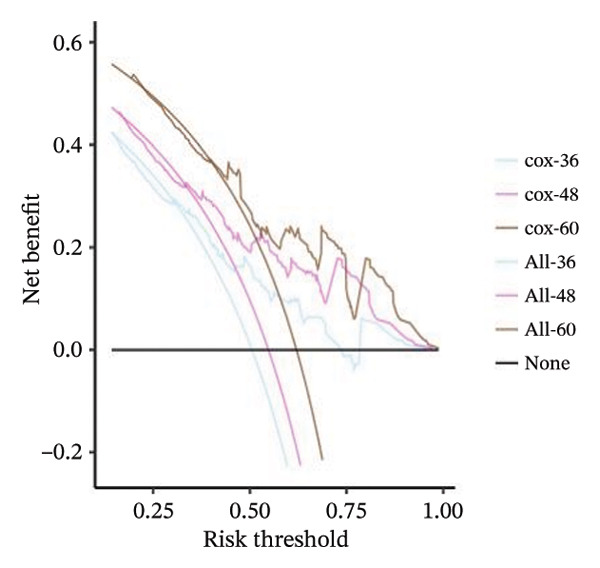
(d)

Comparisons of timeAUC values revealed that the PS‐clinical prediction model outperformed other prediction models based on individual clinical variables such as gender and histologic type, with statistically significant differences in timeAUC values at 36, 48, and 60 months (*p* < 0.05). This indicates the superior predictive efficacy of the combined prediction model (Table S10 in the Supporting Information).

### 3.6. Biological Relevance of the MSH2 Pathomics Model

#### 3.6.1. Differential Gene Expression and Enrichment Analysis

Using the 4‐year SP from the PS‐clinical nomogram (optimal timeAUC = 0.736), patients were dichotomized into high and low SP groups based on the median cutoff (0.473). Differential expression analysis identified 20 genes with significant expression differences, including secreted frizzled related protein 4 (*SFRP4*), lipocalin 2 (*LCN2*), gremlin 1 (*GREM1*), and defensin alpha 6 (*DEFA6*) (Figure 6(a)). Among these, *SFRP4* and *GREM1* were downregulated in the high SP group, whereas *DEFA6* and *LCN2* were upregulated in the high SP group.

FIGURE 6(a) Differential gene analysis between high‐ and low‐ survival‐probability (SP) groups. (b–c) Enrichment analysis of differentially expressed genes between high and low SP groups. A *p* value of less than 0.05 was considered statistically significant. (d) Correlation between SP and immune cell abundance. (e) Tumor mutation burden between high and low SP groups. ns, *p* ≥ 0.05; ^∗^ *p* < 0.05; ^∗∗^ *p* < 0.01; ^∗∗∗^ *p* < 0.001. (f–g) Gene mutations between high and low SP groups: The mutation annotation multi_Hit indicates that genes mutated multiple times within the same sample.

**Figure:**
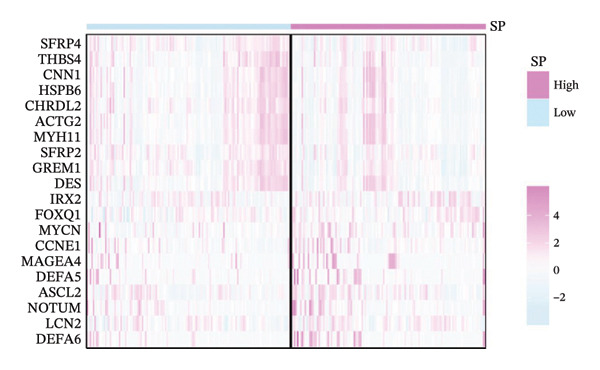
(a)

**Figure:**
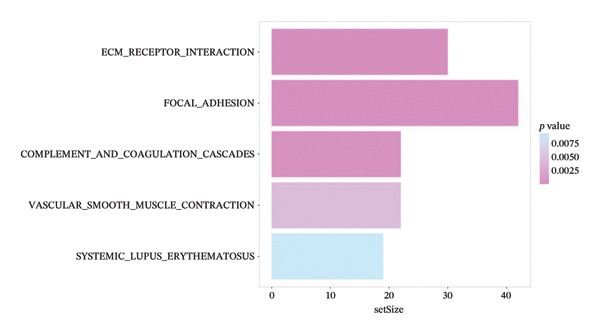
(b)

**Figure:**
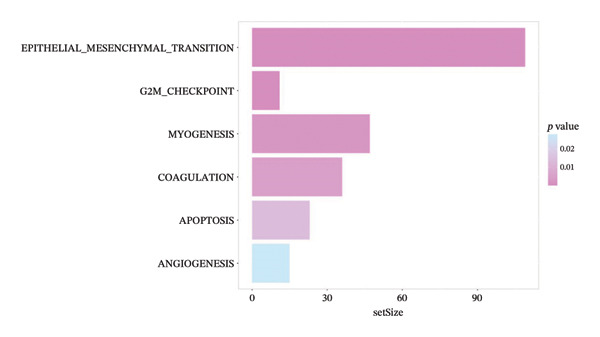
(c)

**Figure:**
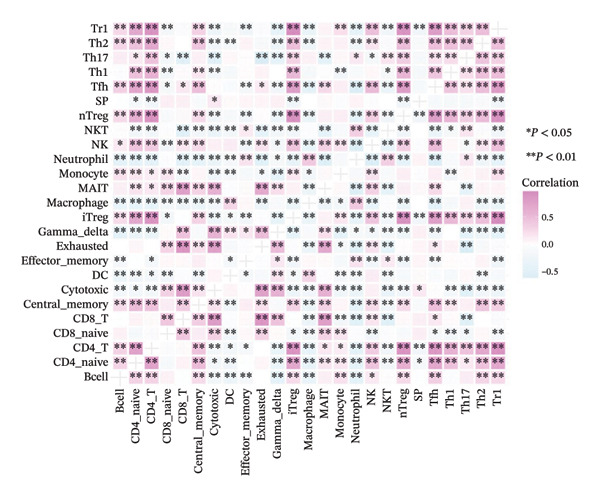
(d)

**Figure:**
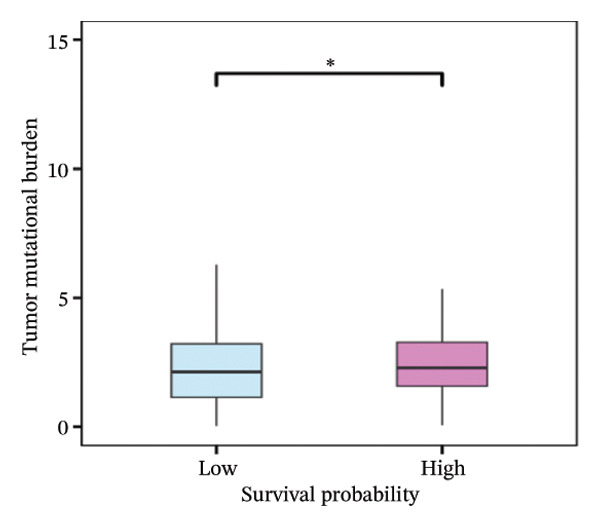
(e)

**Figure:**
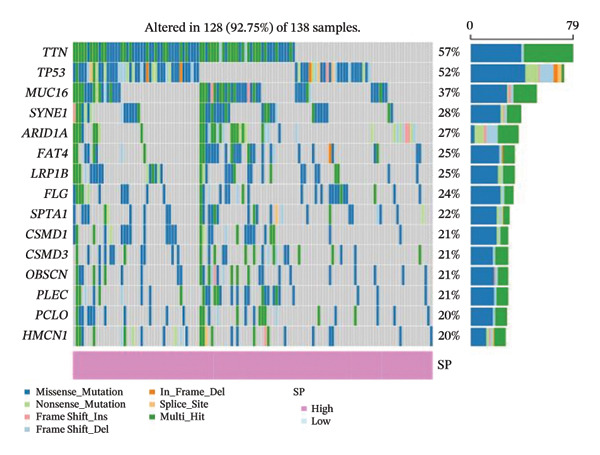
(f)

**Figure:**
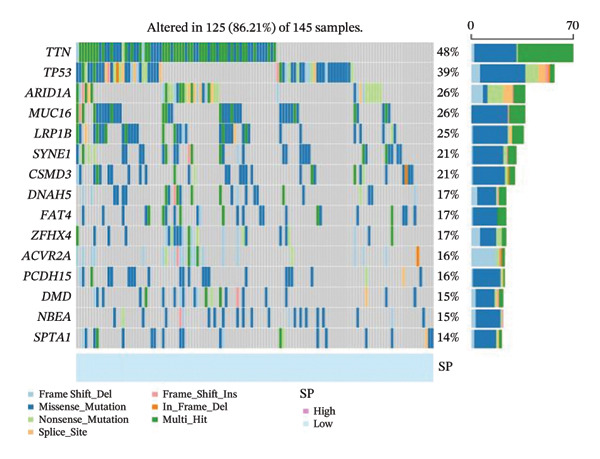
(g)

KEGG pathway enrichment revealed significant involvement of DEGs in five signaling pathways, such as ECM_RECEPTOR_INTERACTION and FOCAL_ADHESION (Figure 6(b)). Hallmark gene set analysis showed enrichment in EPITHELIAL_MESENCHYMAL_TRANSITION and APOPTOSIS pathways (Figure 6(c)).

#### 3.6.2. Immune Cell Infiltration, TMB, and Genetic Variation

Correlation analysis demonstrated that SP negatively correlated with the degree of infiltration of CD4^+^ T cells (*ρ* = −0.212, *p* < 0.01), Tr1 cells (*ρ* = −0.237, *p* < 0.01), nTreg cells (*ρ* = −0.242, *p* < 0.01), iTreg cells (*ρ* = −0.231, *p* < 0.01), and naïve CD4^+^ T cells (*ρ* = −0.145, *p* < 0.05) cells (Figure 6(d)).

Differential analysis revealed that the TMB value of the SP high group was significantly higher than that of the SP low group (*p* < 0.05) (Figure 6(e)). Missense mutations were the most frequent mutation type, followed by frameshift deletions and nonsense mutations. Mutation rates of titin (*TTN*) and tumor protein p53 (*TP53*) exceeded 30% in the high and low SP groups, with higher mutation rates of *TTN*, *TP53*, mucin 16 (*MUC16*), spectrin repeat containing nuclear envelope protein 1 (*SYNE1*), AT‐rich interaction domain 1A (*ARID1A*), FAT atypical cadherin 4 (*FAT4*), and spectrin alpha, erythrocytic 1 (*SPTA1*) observed in the high SP group (Figures 6(f), 6(g)).

## 4. Discussion

Defective MMR is implicated in many cancers [37, 38], but its role in STAD remains incompletely understood [39]. In this study, we integrated MR and pathomics to investigate MMR genes. MR analysis revealed that *MLH1* and *PMS2* were associated with increased GC risk, whereas *MSH2* was linked to decreased risk. Survival analysis confirmed *MSH2* as an independent prognostic factor. We developed an *MSH2*‐based PS for predicting STAD prognosis. An H&E‐based pathomics model accurately predicted *MSH2* expression (AUC = 0.811), and the PS independently served as an independent prognostic factor. Mechanistic analyses connected *MSH2* expression–related genetic instruments were associated with *SFRP4*, CD4^+^ regulatory T‐cell infiltration, *TP53* mutations, and TMB.

*MSH2* encodes a core MMR complex component, forming heterodimers with *MSH6* or *MSH3* to initiate DNA MMR [40]. Elevated *MSH2* expression suppresses genomic instability, tumor heterogeneity, and metastasis. Previous studies have shown consistent positive associations between *MSH2* expression and GC [41, 42]. Furthermore, high *MSH2* expression correlates with improved OS in patients with GC [43], supporting its prognostic and therapeutic relevance. Our findings further reinforce the causal role of *MSH2* in GC pathogenesis and identify it as an independent protective factor for patient prognosis. An interesting divergence was noted for *PMS2*: MR suggested a risk effect, whereas observational survival analysis showed a nonsignificant protective trend. This may stem from fundamental differences between the two approaches: MR infers germline genetic predisposition to disease onset, whereas survival analysis reflects somatic tumor biology and progression within a specific clinical context. The nonsignificant survival trend for *PMS2*, coupled with its inconsistency with the MR signal, suggests that its role in established GC prognosis may be complex or confounded, warranting further investigation in larger cohorts.

Histopathological images capture tumor morphology and microenvironment features predictive of survival [44]. Our pathomics model identified quantitative features (e.g., wavelet_HL_glcm_Correlation and wavelet_HL_firstorder_Mean), reflecting tumor texture and structure at multiple scales, aiding survival prediction. The model’s high accuracy validates AI‐driven histopathology as a clinical tool for personalized treatment. In conclusion, our H&E pathomics model accurately predicted *MSH2* expression (AUC = 0.811, 0.786), which validates the use of AI‐driven histopathological analysis in clinical application and provides a new perspective and important basis for the formulation of individualized treatment strategies. In recent years, radiomics has made significant strides in the field of precision diagnosis of GC, providing a powerful macro‐scale tool for understanding tumor heterogeneity. For example, Gu et al. developed a CT‐based radiomics nomogram that demonstrated excellent performance in differentiating hepatoid adenocarcinoma of the stomach from conventional gastric adenocarcinoma (AUC > 0.9) [45]. This outstanding work demonstrates the value of high‐dimensional features extracted from medical images in addressing the important clinical problem of tumor subtype classification. Our study can meaningfully complement such cutting‐edge radiomics research. While the model by Gu et al. primarily addresses the question of “what”—that is, precise subtype classification of tumors based on imaging features—our pathomics model delves deeper, attempting to explore “why” and “how it functions.” Specifically, it seeks to reveal which core biological functions (*MSH2*‐mediated DNA MMR) are associated with specific histomorphological features and to clarify how this functional state influences the tumor immune microenvironment and genomic stability, ultimately determining patient prognosis. Our pathomics model, derived from routine H&E slides, presents a potential tool for seamless integration into digital pathology workflows. The PS could serve as a quantitative aid to pathologists, potentially flagging cases for more detailed molecular testing or providing adjunctive prognostic information. However, before clinical implementation, rigorous external validation across diverse patient cohorts, scanner platforms, and pathology laboratories is essential to assess its generalizability and robustness. Future prospective studies are needed to evaluate its utility in guiding clinical decision‐making.

Previous studies have indicated that loss of *MSH2* may activate DNA methylation enzymes (e.g., DNA methyltransferase 1 [*DNMT1*]), inducing hypermethylation of *SFRP* family members and forming a methylation feedback loop [46]. *SFRP4* is hypomethylated and overexpressed in GC, linked to enhanced tumor invasiveness [47, 48] and poor patient survival [49]. The tumor immune microenvironment plays a crucial role in tumor development [50]. *MSH2* expression negatively correlated with immune scores and associated with TMB and MSI [42]. Our results suggest that high *MSH2* expression inhibits immunosuppressive Treg infiltration, enhancing anti‐tumor immunity and improving prognosis. Conversely, low *MSH2* promotes Treg infiltration and Pd‐1/PD‐L1 pathway activation, facilitating immune evasion and tumor progression [51]. Conversely, low *MSH2* expression may promote the infiltration of CD4^+^ regulatory T cells (Tregs), including both natural (nTreg) and induced (iTreg) subsets, thereby activating the PD‐1/PD‐L1 immune checkpoint pathway within the tumor microenvironment. This immunosuppressive milieu facilitates tumor immune evasion and promotes cancer progression [52]. As a core component of the DNA MMR process, MSH2 functional loss is associated with high TMB (TMB‐H) in tumors [53, 54]. Studies have reported a significant positive correlation between high *MSH2* gene expression and TMB in GC [41]. Consistent with this, our results demonstrate that TMB in the high SP group, which reflects elevated *MSH2* expression, is observed in *GC* patients. While high TMB is a recognized biomarker for response to immune checkpoint blockade, its association with natural‐history prognosis is not uniform and can vary by cancer type and genomic context. In our cohort, the association between a high SP and higher TMB likely reflects underlying genomic instability, a hallmark of MMRD. This state may generate neoantigens but also co‐occur with aggressive tumor biology. Thus, the prognostic impact of high TMB in this specific molecular subgroup likely depends on the balance between immunogenic potential and other co‐altered oncogenic pathways.

Prior research has identified a negative correlation between microsatellite instability‐high (*MSI-H*) status and *TP53* mutation rates, potentially mediated by DNA methylation patterns and gene activity within the MMR pathway [55]. Considered together, these findings indicate that *MSH2* expression influences genomic stability, modulates the tumor immune microenvironment, and affects mutation profiles of key genes, collectively shaping clinical outcomes in GC. These findings provide novel insights into GC diagnosis and therapeutic targeting.

While computerized H&E‐stained histopathology images show promise in reducing data redundancy and extracting critical morphological features [56], and they support the clinical applicability of our model, several limitations remain. Firstly, the MR findings require further clinical evaluation. Secondly, integrating multiple datasets may introduce batch effects that could affect results consistency. Most importantly, the relatively small size and limited ethnic diversity (predominantly TCGA‐European cohorts) restrict accessibility of the pathomics dataset and the generalizability of our unique findings. We acknowledge that our findings are primarily based on European‐ancestry populations from GWAS and TCGA data. Differences in genetic architecture, allele frequencies, and LD patterns across ethnicities may influence the effect sizes and generalizability of our MR‐derived causal estimates and pathomics model. Therefore, validation in large‐scale, multi‐ethnic cohorts is needed to confirm their broader applicability. Thirdly, the statistical power of our MR analysis is constrained by the sample size of the GC GWAS (633 cases). While we employed stringent instrument selection criteria and sensitivity analyses to ensure robustness, this limited sample size may have reduced our ability to detect causal associations with modest effect sizes. Validation in larger GWAS consortia is warranted.

## 5. Conclusion

In summary, this study is the first to establish, using an integrated MR‐pathomics framework, the dual roles of *MSH2* in STAD as a key regulator of genomic stability and a modulator of the tumor immune microenvironment. We have demonstrated that *MSH2* expression serves as a robust and independent biomarker. Our H&E‐based pathomics model accurately quantifies *MSH2* expression, facilitating precise prognostic stratification and supporting the development of personalized therapeutic strategies for STAD patients.

## Author Contributions

(I) Conception and design: Yingqiao Zhang, Dan Li, Xiushi Zhang, and Yang Liu.

(II) Administrative support: Xiushi Zhang and Yang Liu.

(III) Provision of study materials or patients: Yingqiao Zhang, Dan Li, Wenjuan Zhao, and Pengyu Guo.

(IV) Collection and assembly of data: Yanmeng Liang, Lin Sui, and Yuyao Jin.

(V) Data analysis and interpretation: Ziqi Wang, Xinyu Zhu, Zhenqi Ma, Yanmeng Liang, and Lin Sui.

(VI) Manuscript writing: all authors.

## Funding

This work was supported by the Petrel Research Program at the Harbin Medical University Cancer Hospital, China (No. JJZD2024‐14, to Yang Liu), and the Postdoctoral Scientific Research Developmental Fund of Heilongjiang Province, China (No. LBH‐Q20144, to Yang Liu).

## Disclosure

All authors gave final approval of the manuscript. The authors are accountable for all aspects of the work in ensuring that questions related to the accuracy or integrity of any part of the work are appropriately investigated and resolved.

## Ethics Statement

All original studies obtained informed consent from participants; therefore, this part of the study did not require additional approval from the ethics committee.

## Consent

The authors have nothing to report.

## Conflicts of Interest

The authors declare no conflicts of interest.

## Supporting Information

S. Detailed Methods.

Table S1. SNP information.

Table S2. MR results.

Table S3. Subgroup analysis of *MSH2*.

Table S4. Subgroup analysis of PS.

Table S5. Brief information on GWAS databases in MR studies.

Table S6. Inclusion and exclusion criteria.

Table S7. Baseline data table of gastric cancer patients in the TCGA database.

Table S8. Evaluation indicators of the MSH2 pathomics model.

Table S9. Table of baseline data for each clinical variable grouped by low‐/high‐PS.

Table S10. Comparison of timeAUC values of PS‐clinical prediction models with other prediction models for individual clinical variables.

Figure S1. Flow chart of the MR analysis framework.

Figure S2. Leave‐one‐out plots (A1–A3), funnel plots (B1–B3) and scatter plots (C1–C3) of sensitivity analyses for results of causal association between MMR‐related genes and gastric cancer.

## Supporting information


**Supporting Information** Additional supporting information can be found online in the Supporting Information section.

## Data Availability

All datasets analyzed in this study are publicly accessible from reputable repositories. eQTL data for MMR‐related genes and GWAS data for gastric cancer were obtained from the IEU OpenGWAS project website (https://gwas.mrcieu.ac.uk/). Pathology images of STAD were downloaded from the TCGA database (https://tcga-data.nci.nih.gov/tcga/).
