# Supplementary material for: Integrative Mendelian Randomization and Pathomics Analysis Using Expression Quantitative Trait Loci and Genome‐Wide Association Study Data Identifies Mismatch Repair Genes as Prognostic Biomarkers in Gastric Adenocarcinoma
Source: Int J Genomics. 2026 Mar 9;2026:2686529. doi: 10.1155/ijog/2686529 (PMC12968895; doi:10.1155/ijog/2686529)
Supplement: Supplementary file 1 — Supporting Information Additional supporting information can be found online in the Supporting Information section. [file IJOG-2026-2686529-s001.zip › Supplementary materials.pdf]

Supplementary Materials

S Detailed Methods

S2.1.2 Selection of Instrumental Variables

IVs were selected using a significance threshold of $P<1\times10^{-5}$. To ensure independence and reduce linkage disequilibrium (LD) bias, SNPs were pruned with an LD threshold of $r^2<0.3$ within a 100-kb window. Minor allele frequency (MAF) was set to >0.01 to exclude rare variants. SNPs associated with potential confounders or the outcome variable (GC) were excluded using the GWAS Catalog [1],[2]. IVs within ±300 kb of the cis-acting region of each protein-coding MMR gene based on eQTL data. Palindromic SNPs were removed to avoid strand ambiguity. Anomalous outlier SNPs were identified and removed using Mendelian Randomization Pleiotropy RESidual Sum and Outlier (MR-PRESSO) to minimize bias from horizontal pleiotropy.

S2.1.3 Mendelian Randomization Analysis

The five regression methods used were MR-Egger regression, Inverse Variance Weighted (IVW), Weighted Median, Weighted Mode, and Simple Mode. Because of its efficiency under valid IV assumptions, IVW was the primary method, while the others provided complementary analyses. Cochran's Q-test assessed heterogeneity among SNPs, with $P<0.05$ indicating significant heterogeneity. Horizontal pleiotropy was evaluated using the MR-Egger intercept test and the MR-PRESSO global test; non-significant differences between the intercept term of the MR-Egger regression

and 0 (*P*>0.05) and *P*>0.05 in MR-PRESSO indicate an absence of horizontal pleiotropy.

Sensitivity analysis was performed using the leave-one-out approach, where each SNP was sequentially removed to assess its influence on the overall estimate. All analyses were implemented using the TwoSample MR package in R version 4.1.0, with a significance threshold of α = 0.05.

S*2.2 Survival and Prognostic Analysis of MMR-Related Genes*

OS and prognostic analyses on the positive genes identified through MR were analyzed using the R package survival [3] (R 4.1.0). Kaplan–Meier survival curves were generated to visualize survival rates among different groups (high/low expression) for each positive gene, with the median survival time representing the survival time corresponding to a 50% survival rate. Log-rank tests were used to assess the statistical significance.

Univariate Cox proportional hazards regression was performed to evaluate the association of each positive gene and clinical covariates with OS. Subsequently, multivariate Cox regression was adjusted for potential confounders to identify independent prognostic factors and explore the effect of multiple influences. Hazard ratios (HRs) >1 indicated increased risk, whereas HRs <1 indicated protective effects.

Exploratory subgroup analyses using univariate Cox regression examined the prognostic impact of *MSH2* expression across clinical subgroups (elevated expression group vs. low expression group) according to different covariates. Interaction between

MSH2 expression covariates was assessed using likelihood ratio tests.

S2.3.1 Image acquisition, segmentation and feature extraction

Pathology images of STAD were downloaded from the TCGA (https://tcga-data.nci.nih.gov/tcga/) database (n=443), formalin and paraffin-embedded pathological tissue sections, svs format, with a maximum magnification of 20× or 40× (H&E stained histopathological images (20× or 40× magnification)) [4, 5]. After screening, 291 samples with complete pathological images, gene matrices, and clinical information were finally screened (Table S6 in the supplementary materials).

The tissue regions of the pathological sections were obtained using the OTSU algorithm (https://opencv.org/). The 40× images were divided into multiple 1,024×1,024-pixel subimages; the 20× images were divided into multiple 512×512-pixel subimages and upsampled to 1,024×1,024 pixels. Then, the subimages were reviewed by pathologists to exclude those with poor image quality (contamination, blurriness, or blank areas exceeding 30%). From each pathological image, 20 subimages were randomly selected for subsequent analysis [4, 5]. Employing the open-source package PyRadiomics (https://pyradiomics.readthedocs.io/en/latest/), each sub-image underwent standardization processing, from which 93 original features (including first-order and second-order features) were extracted. Moreover, high-order features (Wavelet (LL, LH, HL, HH)) were also extracted, altogether obtaining 465 features. After extracting

features from 20 sub-images of the pathological image for each patient respectively, the average value was adopted as the pathological omics feature of each sample for subsequent data analysis [6-8].

2.3.2 Establishment and Evaluation of the Random Forest Model

To ensure balanced distribution of clinical variables, the dataset was randomly divided into training (70%) and validation (30%) sets, in a ratio of 7:3. Pathological features (n=465 features) were extracted from the training set using the R package pyradiomics and standardized using z-score normalization via the R package caret, with the mean and standard deviation of the training set applied to standardize the validation set.

Feature selection was performed in two steps. First, the minimum redundancy maximum relevance (mRMR) method identified the top 20 histopathological features; second, recursive feature elimination (RFE) features further refined the feature subset. A random forest (RF) classifier was used to construct a model to predict gene expression levels in the training set.

Model performance was evaluated by accuracy (ACC), specificity (SPE), sensitivity (SEN), positive predictive value (PPV) and negative predictive value (NPV). The area under the receiver operating characteristic (ROC) curve (AUC) quantified overall discrimination. Calibration of the predicted model was evaluated by calibration plots of the calibration curves and the Hosmer–Lemeshow goodness-of-fit test. The Brier score quantified prediction accuracy, with lower scores indicating better consistency. Clinical utility of the pathomics prediction model was evaluated

using DCA.

The model output included a probability pathomics score (PS) predicting gene expression. Differences in PS between high- and low-expression gene groups were compared using the Wilcoxon rank-sum test.

2.2.3 ***Pathomics Score for Survival and Prognosis Analysis***

All statistical analyses were performed using R software (version 4.1.0). The optimal cut-off values for MSH2 and the pathomics score (PS) were determined based on the maximum rank statistics using the survminer package. Baseline clinical characteristics were summarized and compared between the high and low groups: categorical variables (e.g., sex, histological grade, chemotherapy) were analyzed using the chi-square test, while continuous variables (e.g., overall survival [OS] time) were compared using the Wilcoxon rank-sum test. Survival prognosis was evaluated by Kaplan–Meier curves with log-rank tests. Univariate and multivariate Cox proportional hazards models (implemented via the survival package) were employed, with the latter incorporating pre-specified covariates.

Subgroup analyses were stratified by the following clinical covariates: age (≤65 vs. >65 years), sex, pathological stage (I/II vs. III/IV), histological grade (G1/G2 vs. G3), tumor location (antrum/distal, cardia/proximal, fundus/body, gastroesophageal junction), and chemotherapy (yes vs. no). Within each subgroup, a Cox model was used to assess the effect of MSH2 expression level on OS. Interaction effects were tested using the likelihood ratio test. A two-sided p-value < 0.05 was considered statistically significant.

2.3.4 Establishment and Evaluation of the Nomogram Model

Based on clinical data from the TCGA-STAD cohort and pathomics-predicted scores (PS), a nomogram was constructed using Cox proportional hazards regression (via the cph function of the R rms package, v6.7-1) to predict survival probabilities (SP) at 36, 48, and 60 months, incorporating six variables selected through univariate analysis and clinical judgment. The model performance was evaluated in three aspects: First, calibration curves were plotted using bootstrap resampling (B = 1000) to assess the agreement between predicted and observed survival probabilities. Second, time-dependent ROC curves (via the timeROC package, v0.4) were employed to calculate the area under the curve (AUC) and 95% confidence intervals at 36, 48, and 60 months, evaluating the model's discriminative ability and comparing it with univariable models. Finally, decision curve analysis (DCA) (using the ggDCA package, v1.2.0) was conducted to estimate the clinical net benefit across various threshold probabilities. Additionally, patients were stratified into high- and low-risk groups based on the median 4-year survival probability predicted by the nomogram for subsequent comparative analyses. All statistical tests were two-sided, with a significance level set at $p < 0.05$.

2.4.2 Correlation Between Survival Probability and Immune Cell Abundance

RNA-seq expression data (HTSeq-FPKM, log2-transformed) from the TCGA-STAD cohort were uploaded to the ImmuCellAI database (https://guolab.wchscu.cn/ImmuCellAI/) to estimate the relative abundance of

immune cell subsets. To ensure data consistency, sample IDs were truncated to the first 12 characters and matched with the survival probability (SP) information file, followed by removal of irrelevant columns. Spearman's rank correlation analysis was employed to assess the correlation between immune cell abundance and SP, with correlation coefficients (ρ) and P-values reported. Differences in immune cell abundance between high- and low-SP groups were compared using the Wilcoxon rank-sum test. Results were visualized via correlation heatmaps and box plots. A P-value < 0.05 was considered statistically significant.

2.4.3 *Tumor Mutational Burden and Somatic Mutation Analysis*

Somatic mutation data were obtained from the TCGA-STAD cohort, including a tumor mutation burden (TMB) table (TCGA-TMB.txt) and a Mutation Annotation Format (MAF) file (TCGA-STAD_SNP.maf). Data processing was performed using the R package maftools (v2.14.0). Sample barcodes were uniformly truncated to the first 12 characters to ensure consistency with clinical data. For the TMB table, samples from the STAD project were first selected, irrelevant columns were removed, and duplicate samples were averaged before merging into the survival probability (SP) information file. For TMB analysis, the number of all nonsynonymous somatic mutations was calculated using the built-in workflow of maftools and normalized by exome size to yield the number of mutations per megabase (Mb). The TMB metric used was total_perMB (mutations per Mb). Differences in TMB between high- and low-SP groups were compared using the Wilcoxon rank-sum test, with median values

and P-values reported. For mutational profile analysis, the MAF file was stratified by SP group, and oncoplots were generated via maftools to display the top 15 most frequently mutated genes in the TCGA-STAD cohort. Different mutation types were color-coded, and clinical annotations (SP group) were included in the plot for comparison. A P-value < 0.05 was considered statistically significant.

Table S5: Brief information on GWAS databases in MR Studies

Data source	Phenotype	Sample size	Cases	Population	Adjustment
eqtl-a-ENSG00000095002	MSH2	31644	-	European	Males and Females
eqtl-a-ENSG00000076242	MLH1	14263	-	European	Males and Females
eqtl-a-ENSG00000119684	MLH3	31684	-	European	Males and Females
eqtl-a-ENSG00000113318	MSH3	14263	-	European	Males and Females
eqtl-a-ENSG00000116062	MSH6	14263	-	European	Males and Females
eqtl-a-ENSG00000064933	PMS1	14263	-	European	Males and Females
eqtl-a-ENSG00000122512	PMS2	31470	-	European	Males and Females
finn-b-C3_STOMACH_EXALLC	Malignant neoplasm of stomach (all cancers excluded)	174639	633	European	Males and Females

Table S6: Inclusion and exclusion criteria

TCGA-STAD clinical data<br>Stomach adenocarcinoma	Number of samples excluded	Number of samples excluded
Total number of cases	-	443
Samples from primary and first diagnosed stomach adenocarcinoma were screened	3	440
Those with missing survival data and less than 1 month of survival were excluded	49	391
Samples with missing data on age, tumor stage, pathological grade, and anatomic neoplasm division were excluded	48	343
Samples with RNA-seq were selected	15	328
TCGA-STAD pathological images	Number of samples excluded	Number of samples excluded
Total number of cases	-	372
Samples with substandard image quality were excluded	-	372
Intersected samples with clinical data and RNA-seq were screened	81	291

Table S7: Baseline data table of gastric cancer patients in TCGA database

Variables	Total (n=328)	Low (n=161)	High (n=167)	*p*
Age, n (%)				0.049
-65	152(46)	84(52)	68(41)	
66-	176(54)	77(48)	99 (59)	
Gender, n (%)				0.661
Female	119(36)	56(35)	63(38)	
Male	209(64)	105(65)	104(62)	
Pathologic _ stage, n (%)				0.637
I/II	150(46)	71(44)	79(47)	
III/IV	178(54)	90 (56)	88(53)	
Histologic _ grade, n (%)				0.263
G1/G2	121(37)	54(34)	67(40)	
G3	207(63)	107(66)	100(60)	
Tumor _ location, n (%)				0.235
Antrum/Dista	124(38)	63(39)	61(37)	
Cardia/Proximal	48(15)	28(17)	20(12)	
Fundus/Body/Gastroesophageal Junction	156(48)	70(43)	86 (51)	
Chemotherapy, n (%)				0.841
NO	174(53)	84 (52)	90(54)	
YES	154(47)	77(48)	77(46)	

Table S8: Evaluation indicators of MSH2 pathomics model

name	train_auc	train_ci	Train_thre	Train_acc	Train_sens	Train_spec	Train_ppv	Train_npv	train_brierScore
MSH2_cat_PS	0.811	0.753-0.87	0.495	0.755	0.815	0.688	0.746	0.767	0.194
name	validation_auc	validation_ci		validation_acc	validation_sens	validation_spec	validation_ppv	validation_npv	validation_brierScore
MSH2_cat_PS	0.786	0.688-0.883		0.713	0.565	0.878	0.839	0.643	0.205

Table S9: Table of baseline data for each clinical variable grouped by Low/High PS

Variables	Total (n=291)	Low (n=165)	High (n=126)	*p*
Age, n (%)				1
-65	135(46)	77(47)	58(46)	
66-	156(54)	88(53)	68(54)	
Gender, n (%)				1
Female	106(36)	60(36)	46(37)	
Male	185(64)	105(64)	80(63)	
Pathologic _ stage, n (%)				0.907
I/II	127(44)	73(44)	54(43)	
III/IV	164(56)	92(56)	72(57)	
Histologic _ type, n (%)				0.002
Adenocarcinoma, Intestina	139(48)	64(39)	75(60)	
Adenocarcinoma, NOS	92(32)	63(38)	29(23)	
Adenocarcinoma, Others	60(21)	38(23)	22(17)	
Tumor _ location, n (%)				0.937
Antrum/Distal	117(40)	65(39)	52(41)	
Cardia/Proxima	41(14)	24(15)	17(13)	
Fundus/Body/Gastroesophageal Junction	133(46)	76(46)	57(45)	
Chemotherapy, n (%)				0.183
NO	155(53)	94(57)	61(48)	

YES	136(47)	71(43)	65(52)

Table S10: Comparison of timeAUC values of PS-clinical prediction models with other prediction models for individual clinical variables

timeAUC	PS-Clinical	PS	Age	Gender	Pathologic_stage	Histologic_type	Tumor_location	Chemotherapy
t=36	0.684	0.559	0.595	0.467	0.566	0.54	0.597	0.54
t=48	0.736	0.637	0.568	0.456	0.584	0.585	0.459	0.613
t=60	0.719	0.617	0.612	0.501	0.603	0.448	0.449	0.615

P value	PS-Clinical vs PS	PS-Clinical vs Age	PS-Clinical vs Gender	PS-Clinical vs Pathologic_stage	PS-Clinical vs Histologic_type	PS-Clinical vs Tumor_location	PS-Clinical vs Chemotherapy
t=36	0.010683783	0.08942417	0.000157861	0.010245656	0.019063513	0.170644601	0.016256457
t=48	0.146025529	0.012968955	0.000249065	0.020627498	0.042223538	0.000832671	0.129282417
t=60	0.294158662	0.157201834	0.032159489	0.206099535	0.002581832	0.001083468	0.317129646

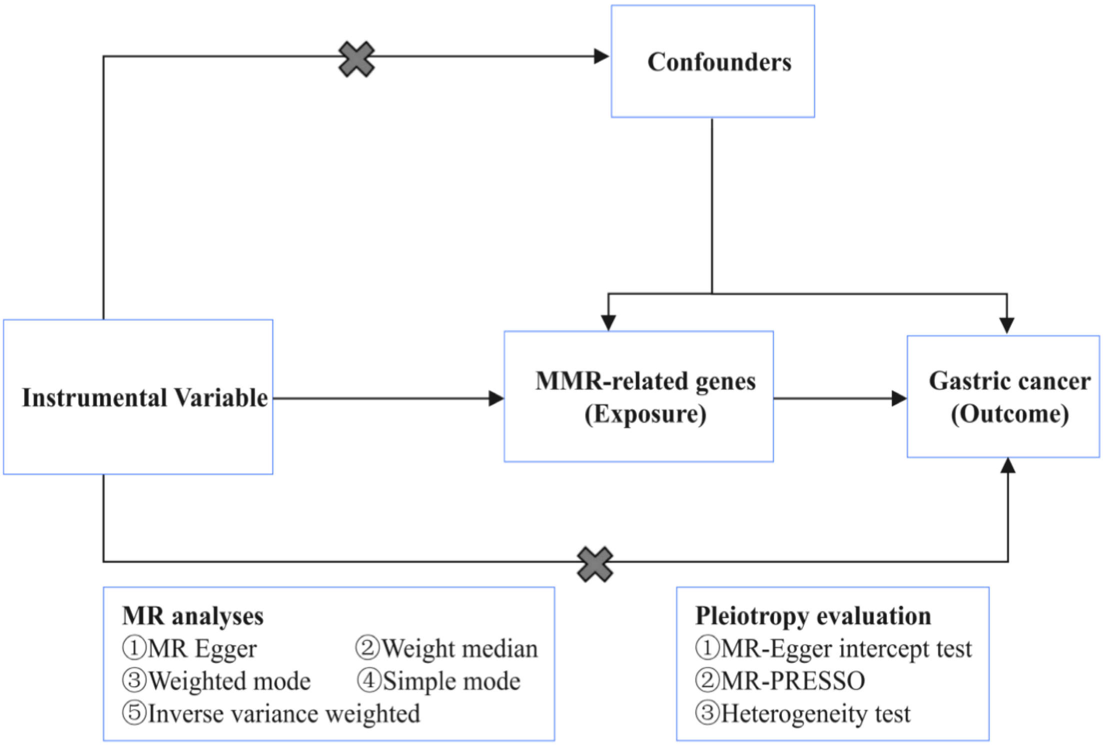

Fig. S1: Flow chart of the MR Analysis framework

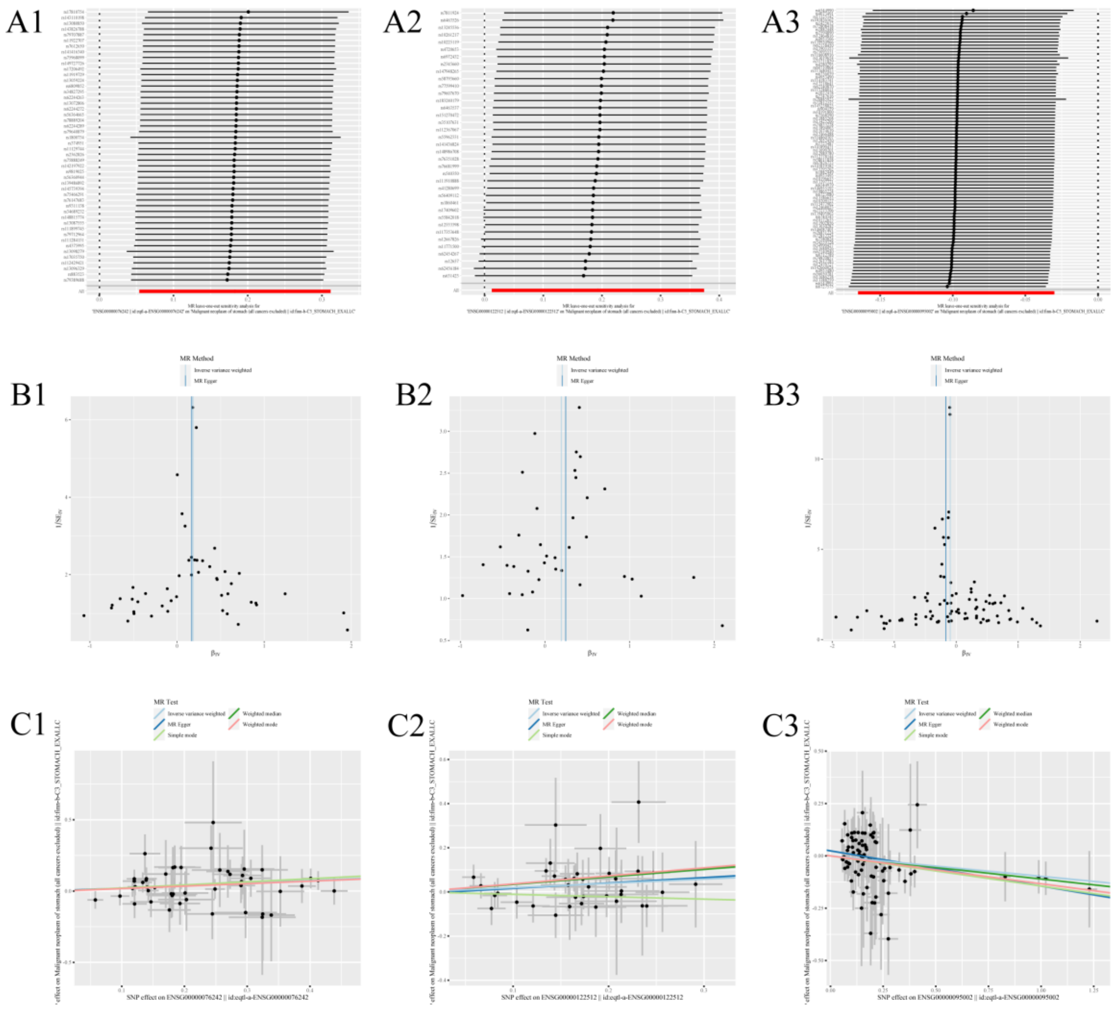

Fig. S2: Leave-one-out plots (A1-A3), funnel plots (B1-B3) and scatter plots (C1-C3) of sensitivity analyses for results of causal association between MMR-related genes and gastric cancer

References

1. Sollis E, Mosaku A, Abid A, Buniello A, Cerezo M, Gil L, et al. The NHGRI-EBI GWAS Catalog: knowledgebase and deposition resource. Nucleic Acids Res. 2023;51(D1):D977-d85. https://doi.org/10.1093/nar/gkac1010.
2. Machlowska J, Baj J, Sitarz M, Maciejewski R, Sitarz R. Gastric Cancer: Epidemiology, Risk Factors, Classification, Genomic Characteristics and Treatment Strategies. Int J Mol Sci. 2020;21(11). https://doi.org/10.3390/ijms21114012.
3. Rizvi AA, Karaesmen E, Morgan M, Preus L, Wang J, Sovic M, et al. gwasurvivr: an R package for genome-wide survival analysis. Bioinformatics. 2019;35(11):1968-70. https://doi.org/10.1093/bioinformatics/bty920.
4. Chen L, Zeng H, Zhang M, Luo Y, Ma X. Histopathological image and gene expression pattern analysis for predicting molecular features and prognosis of head and neck squamous cell carcinoma. Cancer Med. 2021;10(13):4615-28. https://doi.org/10.1002/cam4.3965.
5. Zeng H, Chen L, Zhang M, Luo Y, Ma X. Integration of histopathological images and multi-dimensional omics analyses predicts molecular features and prognosis in high-grade serous ovarian cancer. Gynecol Oncol. 2021;163(1):171-80. https://doi.org/10.1016/j.ygyno.2021.07.015.
6. Saednia K, Lagree A, Alera MA, Fleshner L, Shiner A, Law E, et al. Quantitative digital histopathology and machine learning to predict pathological complete response to chemotherapy in breast cancer patients

using pre-treatment tumor biopsies. Sci Rep. 2022;12(1):9690. https://doi.org/10.1038/s41598-022-13917-4.

7. Liu K, Hu J. Classification of acute myeloid leukemia M1 and M2 subtypes using machine learning. Comput Biol Med. 2022;147:105741. https://doi.org/10.1016/j.compbiomed.2022.105741.

8. Nishio M, Nishio M, Jimbo N, Nakane K. Homology-Based Image Processing for Automatic Classification of Histopathological Images of Lung Tissue. Cancers (Basel). 2021;13(6). https://doi.org/10.3390/cancers13061192.
